# Potential Therapeutic Approaches through Modulating the Autophagy Process for Skin Barrier Dysfunction

**DOI:** 10.3390/ijms22157869

**Published:** 2021-07-23

**Authors:** Min Sik Choi, Yoon-Jee Chae, Ji Woong Choi, Ji-Eun Chang

**Affiliations:** 1 Lab of Pharmacology, College of Pharmacy, Dongduk Women’s University, Seoul 02748, Korea; mschoi@dongduk.ac.kr; 2 College of Pharmacy, Woosuk University, Wanju-gun 55338, Korea; yjchae@woosuk.ac.kr; 3 College of Pharmacy, Gachon University, Incheon 21936, Korea; pharmchoi@gachon.ac.kr; 4 Lab of Pharmaceutics, College of Pharmacy, Dongduk Women’s University, Seoul 02748, Korea

**Keywords:** autophagy, skin barrier dysfunction, psoriasis, vitiligo, infectious skin diseases, skin cancer, acne, skin aging

## Abstract

Autophagy is an attractive process to researchers who are seeking novel potential treatments for various diseases. Autophagy plays a critical role in degrading damaged cellular organelles, supporting normal cell development, and maintaining cellular homeostasis. Because of the various effects of autophagy, recent human genome research has focused on evaluating the relationship between autophagy and a wide variety of diseases, such as autoimmune diseases, cancers, and inflammatory diseases. The skin is the largest organ in the body and provides the first line of defense against environmental hazards, including UV damage, chemical toxins, injuries, oxidative stress, and microorganisms. Autophagy takes part in endogenous defense mechanisms by controlling skin homeostasis. In this manner, regulating autophagy might contribute to the treatment of skin barrier dysfunctions. Various studies are ongoing to elucidate the association between autophagy and skin-related diseases in order to find potential therapeutic approaches. However, little evidence has been gathered about the relationship between autophagy and the skin. In this review, we highlight the previous findings of autophagy and skin barrier disorders and suggest potential therapeutic strategies. The recent research regarding autophagy in acne and skin aging is also discussed.

## 1. Introduction

The complex term ‘autophagy’ is derived from the Greek word ‘self-eating’, with ‘auto’ meaning ‘self’ and ‘phagy’ meaning ‘eat’ [1]. Autophagy is a major intracellular degradation process for damaged and dysfunctional organelles to protect cells under high-stress conditions, such as deficiency of nutrients, growth factors loss, oxidative stress, and hypoxia [2,3,4]. It is also involved in providing nutrients and energy, which are essential for normal cell development under stress [1]. In addition, it plays a crucial role in maintaining cellular homeostasis by selectively clearing surplus or damaged organelles, proteins, and pathogens [5].

The skin comprises the largest portion of the body and provides a first-line barrier function against environmental dangers, including ultraviolet (UV) radiation, pathogens, allergens, injuries, oxidative stress, and toxic chemicals [6,7]. The skin works as more than just a physical barrier; it serves as an active immune organ [8]. It also prevents dehydration, controls body temperature, and has self-healing abilities. In addition, the skin is a sensory organ that provides the central nervous system with pain, thermal, and touch information [9]. Normal skin is composed of three main layers, which are the epidermis, dermis, and hypodermis [10]. The epidermis is the outermost part of the skin and provides a barrier function, innate immunity, and a protective effect from UV radiation. Keratinocytes, melanocytes, and Langerhans cells comprise a major portion of the epidermis [11]. Keratinocytes differentiate into three layers called the stratum spinosum, stratum granulosum, and stratum corneum (SC). The SC provides the main barrier function in the outer layer of the epidermis. The dermis includes fibroblasts, mast cells, macrophages, and circulating immune cells. It is associated with skin elasticity, thermal control, and wound healing. The hypodermis is the deepest layer of the skin and is mainly composed of adipocytes. It insulates the body, protects against injuries, and reserves energy supply [12].

Autophagy participates in the endogenous defense mechanism by regulating skin homeostasis, causing it to play a pivotal role in the development and progression of skin-related diseases [13]. Understanding the association between autophagy and skin barrier dysfunction could provide clues for finding novel potential therapeutic approaches for various skin diseases.

## 2. Mechanism of Autophagy

Autophagy is the biological process by which the constituents of a cell break down in autolysosomes [14,15]. The clarification of the crucial genes involved in autophagy has accelerated our understanding of the pathophysiology of human diseases. Particularly, in terms of experimental techniques, mutations in autophagy-related genes (ATGs) are now being used to discover new therapeutic targets in the autophagy pathway for various human diseases [16].

Categorized by the method of transporting cargo into lysosomes, three different types of autophagy are well-known so far [17]. These are macroautophagy, microautophagy, and chaperone-mediated autophagy (CMA). Macroautophagy is the major form of autophagy that reacts to physiological or pathological stimuli. Microautophagy is related to the direct engulfing of cytoplasmic constituents by lysosomes [18], while chaperone-mediated autophagy is linked to the translocation of substrate molecules through the lysosomal membrane [19].

During macroautophagy (hereafter referred to as autophagy), a part of the cytoplasm is enclosed by a double-membrane organelle called an autophagosome. After fusion of the outer autophagosomal membrane and the lysosomal membrane occurs, lysosomal enzymes break down the inner membrane of the autophagosomes and the enclosed intracellular materials. In fact, autophagy was initially recognized as a non-selective biological process, but now it is known to break down specific materials, (e.g., damaged lysosomes, impaired mitochondria, or intracellular microbes) and each of these autophagy processes now has its own name (e.g., lysophagy, mitophagy, and xenophagy, respectively) [16,20,21].

The autophagy process in cells, which proceeds by the above molecular mechanisms, can be broadly grouped into the following two functions. First, autophagy plays a role in adapting to metabolic needs. During fasting or aerobic exercise, for example, autophagy is upregulated and breaks down macromolecules into smaller ones to be used as an energy source or a building block for other biomolecules [22]. In addition, autophagy is known to be important for the development, growth, and differentiation of various living tissues [23]. These autophagic functions related to energy metabolism appear mainly through the regulation of energy sensors, AMP-activated protein kinase (AMPK), and mTOR (mechanistic target of rapamycin). Among the skin barrier dysfunction-related diseases considered in this review, vitiligo and skin aging-related diseases fall into this category. The detailed mechanisms will be dealt with in each section.

The second role of autophagy is related to intracellular homeostasis. Although it is primarily a process of removing old or waste substances from cells to prevent them from accumulating, it has been reported that autophagy plays an important role in maintaining homeostasis in the immune response or inflammatory process [15,24]. Autophagy is known to allow the host to activate the immune system, thereby regulating the state of infection and reducing uncontrolled inflammation [25]. The autophagy function in this respect can be found in the process of skin barrier dysfunction in various infectious diseases that invade the skin. It is also found in psoriasis associated with various microbial and viral infections, or acne conditions requiring the removal of sebocyte debris.

As the mechanism of autophagy has been uncovered in many contexts, it is believed that autophagy is closely related to human disease. However, little evidence has been gathered that autophagy increases or decreases under certain conditions, as human autophagy activity cannot be accurately measured. Recent human genome research results further increase the possibility of measuring autophagy in inflammatory diseases, cancers, and autoimmune diseases [26,27,28].

## 3. Autophagy in Autoimmune Skin Disorders

### 3.1. Psoriasis

Psoriasis is a chronic autoimmune T cell-mediated skin disorder characterized by heavily scaled red or salmon pink plaques [29]. Sustained inflammation and uncontrolled keratinocyte proliferation are well-known pathological features of psoriasis [30,31]. Around 2–4% of the world population has psoriasis [32]; however, the prevalence varies by race, age, and gender [33]. Lee et al. demonstrated a major role of autophagy in keratinocyte inflammation regulation [34]. In the study, keratinocyte autophagy downregulated the expression of scaffolding adaptor protein p62/SQSTM1 (p62), leading to decreased inflammatory cytokine production and keratinocyte proliferation.

Several bacteria, fungi, and viruses have been linked to the development of psoriasis [35]. Among them, Streptococcus pyogenes tonsillar infection is the most prominent factor leading to the triggering and aggravation of psoriatic symptoms [36]. As autophagy promotes bacterial clearance, decreased autophagy in psoriasis may attenuate both clearance and immune response to bacteria [37].

Douroudis et al. reported a possible impact of polymorphisms in the ATG16L1 gene (rs10210302, rs12994971, rs2241880, rs2241879, and rs13005285) on psoriasis susceptibility [38]. ATG16L1 is essential for autophagy [39]. Therefore, decreased ATG16L1 affects the autophagy machinery, resulting in cell death, tissue damage, and chronic inflammation [40].

Many researchers demonstrated that both T helper 17 (Th17) cells and regulatory T (Treg) cells play pivotal roles in the pathogenesis of psoriasis [41,42,43]. Th17 cells produce interleukin-17A (IL-17A), leading to the stimulation of keratinocyte proliferation and exacerbating skin inflammation [44,45]. A high level of Th17 was reported in patients with psoriasis and was correlated with the clinical severity and activity of the disease [46,47]. Treg cells are the key players in maintaining skin immune homeostasis and preventing autoimmune disease by suppressing the immune response [48]. However, the suppressive function of Tregs is impeded in patients with psoriasis, causing an imbalance of Th17 and Treg cells and exacerbating the disease [49,50,51]. Autophagy was improved when Th17 was decreased and Treg was increased by metformin treatment [52]. Since the ratio of Th17 to Treg was increased in the setting of psoriasis, the upregulation of autophagy may have potential as a novel treatment for psoriasis.

### 3.2. Vitiligo

Vitiligo is the most common pigmentation disorder, with a worldwide prevalence of around 1% of the population [53,54]. It is an acquired autoimmune disease characterized by patchy white skin caused by the loss of CD8+ T cell-mediated melanocytes from the epidermis [55,56,57]. Vitiligo is classified into two groups: segmental vitiligo and non-segmental vitiligo (NSV) [58]. Segmental vitiligo shows localized partial loss of melanin, while NSV corresponds to generalized melanocyte loss caused by the autoimmune response [59].

According to previous reports, cellular stress may induce autophagy, promote proinflammatory heat shock proteins 70 (HSP70) and antigen-carrying exosome release, and induce X-box binding protein 1 (XBP1)-dependent IL-6 and IL-8 secretion [60,61]. In the setting of vitiligo, patients show higher stress levels compared with healthy controls [60].

In addition, autophagy is known to regulate melanosome degradation [62,63], promoting the survival and proliferation of melanocytes [13]. Jeong et al. suggested a possible association between the UV radiation resistance-associated gene (UVRAG) polymorphisms (rs1458836 and rs7933235) and NSV susceptibility in a Korean sample [64], and another group led by Jeong reported a relationship between Granzyme B (GZMB) polymorphisms (rs2236338, rs11539752, rs10909625, and rs8192917) and NSV development in a Korean population [59], supporting the theory that autophagy is essential for cellular homeostasis.

Taken together, inducing autophagy may provide a potential therapeutic option for vitiligo. In a recent study, Bastonini et al. demonstrated that induction of autophagy plays a protective role against intrinsic metabolic stress and enhances survival in vitiligo [54].

## 4. Autophagy in Infectious Skin Diseases

### 4.1. Group A Streptococcus

One of the most studied skin pathogens associated with autophagy is Group A *Streptococcus* (GAS, *Streptococcus pyogenes*). GAS is a Gram-positive species and a beta-hemolytic coccus [65]. It is associated with a wide range of acute and chronic diseases, including pharyngitis, streptococcal toxic shock syndrome, and acute post-streptococcal glomerulonephritis (APSGN) [66]. In addition, GAS induces a wide range of skin diseases, which include impetigo, psoriasis, and the immune-mediated diseases of acute rheumatic fever (ARF) [67,68]. Autophagy may be a critical mechanism to eliminate GAS in the skin, as evidenced by various studies. Nakagawa et al. first demonstrated that GAS is effectively eliminated via the autophagy-mediated pathway [69]. They revealed that the amount of microtubule-associated protein light chain 3 (LC3)-II, which is a marker for the formation of autophagosomes, increased following GAS infection, suggesting that GAS infection induces autophagy. This study also supported this claim by showing that LC3-II formation and LC3-positive autophagosome were not observed in ATG5-deficient cells. Adaptors such as p62 [70], nuclear dot protein 52 (NDP52) [71,72], and neighbor of BRCA1 gene 1 (NBR1) [73] are essential in recognizing GAS after it is flagged with polyubiquitinated protein and can recruit LC3. Furthermore, it was reported that insufficient acidification of autophagosomes permits GAS to replicate, leading to the growth of GAS in endothelial cells [74], which implies a significant role of low pH in GAS removal by autophagy. Moreover, recent studies have suggested crucial mechanisms of autophagy-mediated removal of GAS within cells [75,76,77], indicating that the regulation of autophagy can lead to therapeutic benefits in infectious diseases caused by GAS.

Interestingly, multiple evasion mechanisms of GAS have been reported to avoid degradation via autophagy. For example, expression of streptococcal pyrogenic exotoxin B (SpeB1), a streptococcal cysteine protease, is observed in the globally disseminated M1T1 clone of GAS and is known to induce the degradation of p62, NDP52, and NBR1, resulting in the escape of GAS from host autophagy [78].

### 4.2. Herpes Simplex Virus

Autophagy also plays an important role in skin infections caused by viruses. Herpes simplex virus (HSV) is commonly observed worldwide and highly contagious [79]. It shows a distinctive structure, consisting of a DNA-filled capsid, an envelope with lipids, and a proteinaceous tegument layer [80], and is categorized into two groups: HSV-1, which induces herpes labialis, pharyngitis, and keratitis, and HSV-2, which induces common genital herpes [81]. The regulation of autophagy by HSV-1 may be cell type-dependent, as reported in previous studies; however, most studies have concluded that autophagy is involved in reducing HSV-1 replication and reproduction [82]. It was reported that HSV-1 induces the formation of four-layered membrane structures from the nuclear envelope, which have autophagosome-like structures, and evidence suggests that the structures were fused with lytic organelles [83,84]. In addition, autophagy induced during HSV-1 infection triggers a vacuolar response, which increases the processing and presentation of the peptide HSV-1 glycoprotein B on major histocompatibility complex (MHC) class I molecules [83]. In contrast to HSV-1, little is known about the roles of autophagy in HSV-2 infection. It was reported that the removal of ATG5 function by gene knockdown decreased the processing and presentation of HSV-2 antigen on MHC class II molecules [85], but further study is necessary to elucidate the specific roles of autophagy in HSV-2 within the cells.

Like other pathogens, HSV can express various proteins that counter autophagy. The first anti-autophagic protein discovered was infected cell protein 34.5 (ICP34.5), encoded by HSV-1 [86]. ICP34.5 binds to and inhibits beclin 1, which is an essential protein for the autophagy process, and the absence of ICP34.5 facilitates autophagy by activating the eukaryotic translation initiation factor 2-α kinase 2 (EIF2AK2/PKR) pathway. In addition, Us11 encoded by HSV-1 was determined to be an anti-autophagy protein that directly binds to PKR [87].

### 4.3. Candida Albicans

*Candida albicans* (*C. albicans*) is a commensal fungus commonly causing opportunistic infections of the skin, mucosa, and reproductive tract. It normally causes no harm; however, it sometimes causes life-threatening diseases in immunocompromised patients. The involvement of the autophagy process in the removal of *C. albicans* within the cells was suggested in a study by Nicola et al. [88]. They confirmed the existence of LC3 in most macrophage vacuoles containing *C. albicans* and observed that removing ATG5 function by RNA interference decreased the phagocytosis of C. albicans. Thus, the findings regarding the mechanisms of autophagy in *C. albicans* may facilitate the identification of promising therapeutic targets. In this context, Zhang et al. reported that the V-ATPase subunit VMA5 is associated with autophagy completion for *C. albicans* and hyphal development. In addition, inositol polyphosphate kinase Vip1 has been reported as an important factor in the autophagy of *C. albicans* [89], enriching our knowledge and understanding of autophagy and its role in *C. albicans* infections.

## 5. Autophagy in Skin Cancer Diseases

The role of autophagy in cancer is complicated; it may lead to tumor survival or tumor death, which are opposing consequences. In the early stages of cancer, autophagy may act as a tumor suppressor by preventing chronic tissue damage and cancer initiation [90,91,92]. On the other hand, autophagy works as a tumor promotor in established cancers by supporting metabolism, tumorigenesis, and survival [90,91,92]. Many researchers have investigated the relationship between autophagy and various cancers in an attempt to discover a potential therapeutic approach for these diseases.

### 5.1. Skin Squamous Cell Carcinoma

Skin squamous cell carcinoma (SSCC) is the second most common skin cancer, and its incidence has continued to increase worldwide over the past decades [93,94]. SSCC development is known to be mainly related to chronic, cumulative UV exposure and immunosuppression [93,95].

Verschooten et al. reported that blocking autophagy using chloroquine-enhanced luteolin induced apoptosis in metastatic squamous cell carcinoma cells [96]. Ou et al. also demonstrated that the inhibition of protective autophagy by chloroquine could promote apoptosis induced by gefitinib, a selective epidermal growth factor receptor (EGFR) tyrosine kinase inhibitor, in SSCC [97]. Chloroquine effectively suppressed the proliferation, migration, and invasiveness of SSCC while enhancing apoptosis. The blockade of autophagy by chloroquine was demonstrated by the increased cleavage of caspase-3 and the accumulation of LC3-II and SQSTM1 (p62). A similar study was performed by Wang’s group and concluded that chloroquine exhibits a synergetic apoptotic effect mediated by gefitinib in SSCC cells [98]. The suppression of autophagy by chloroquine was demonstrated by the alteration of LC3-II.

Zhang et al. demonstrated that when SSCC cells were treated with 3-methyladenine (3-MA), an autophagy inhibitor, followed by 5-fluorouracil (5-FU), the inhibition of proliferation, migration, and invasion of SSCC cells was enhanced; furthermore, the apoptosis of the cells also increased [99]. In this study, autophagy in SSCC was confirmed, as the expression of the autophagy-related gene LC3 showed a negative correlation with Bcl2 and/or survival.

Overall, the above research suggests the potential of autophagy as a target for SSCC treatment. In addition, a combination strategy of autophagy inhibitors and anti-cancer agents may be an effective novel treatment option for SSCC using autophagy regulation.

### 5.2. Melanoma

Melanoma arises from the transformation of melanocytes, which produce pigments [100]. It is one of the most lethal tumors, and the overall mortality rate has been increasing [101]. Melanoma is characterized by a high capability of invasion and rapid metastatic potential, which leads to a poor prognosis of metastatic melanoma [102]. Around 50% of melanoma patients show BRAF mutations, which lead to dysregulated downstream activation of the MEK and ERK pathways [103]. Mutated BRAF genes are associated with the upregulation of proliferation, differentiation, survival, invasion, and angiogenesis of melanoma [104]. The currently approved therapies for metastatic melanoma include not only BRAF and MEK inhibitors but also immune checkpoint inhibitors such as anti-CTLA-4 and anti-PD-1 [101]. However, novel strategies are needed to overcome the existing drug resistance and tumor recurrence of the current treatments [105,106].

High levels of autophagy in melanoma assessed by LC3B were revealed to be associated with tumor cell proliferation, metastasis, and poor outcomes [107]. From this point of view, many researchers have examined autophagy inhibition strategies as potential treatments for melanoma. Xie et al. discovered that treatment with dabrafenib, a BRAF inhibitor, increased the anti-tumor effect in a melanoma mouse model [108].

Chloroquine is also known to mediate autophagy inhibition in melanoma [109,110]. Since hypoxia-inducible factor-1α (HIF-1α) allows cell growth under metabolic stress and hypoxia, Egger’s team hypothesized that a combination of an HIF-1α inhibitor and chloroquine would have an anti-tumor effect on melanoma cells [111]. As expected, combining the HIF-1α inhibitor echinomycin with chloroquine improved melanoma cytotoxicity under hypoxic conditions.

All these findings support the hypothesis that targeting autophagy is a promising clinical approach in melanoma therapy. In recent years, clinical trials that involve blocking chloroquine-mediated autophagy have been initiated in various types of cancers, including melanoma [112,113].

## 6. Autophagy in Other Skin Diseases

In addition to the above-mentioned skin diseases, the possibility of its association with autophagy in several diseases has been suggested. Acne and skin aging, which are discussed in this section, contain pathological mechanisms associated with damage to the skin barrier. Using the latest research papers on each disease, the possibility of treatment and prevention of skin diseases through the control of autophagy will be discussed.

### 6.1. Acne

As mentioned earlier, autophagy is a catabolic process that discards impaired organelles to preserve cellular homeostasis. From this point of view, it can be assumed that autophagy is also involved in sebaceous gland homeostasis. Here, two scientific views on the relationship between acne and autophagy will be reviewed in terms of damage and repair of the skin barrier.

As an exocrine organ present in the skin, the sebaceous glands are involved in the progression of a skin disease known as acne vulgaris. Acne occurs when the outlet from the gland to the surface of the skin is plugged, allowing sebum to accumulate in the follicle and sebaceous duct [114]. The sebaceous glands are made up of sebocytes in proliferating, maturating, or matured states. The excessively generated sebum stimulates the hair follicle to eliminate these sebocytes, and the removed cells clump to block the sebaceous duct, forming comedones and thereby inducing the acne inflammatory response. Through the subsequent inflammatory reaction, the breakdown of triglycerides in the sebum occurs and creates the typical lesions of acne by releasing free fatty acids. Considering this background, the formation of microcomedones and open/closed comedones is induced by the leakage and accumulation of cell debris from incompletely removed sebocytes into the sebaceous duct. Interestingly, it has been reported that the process of eliminating sebocytes in the sebaceous glands is followed by autophagy [115]. This process seems to be aimed at providing a substrate for energy generation and biosynthesis of cellular proteins for normal sebaceous gland function. According to the results of the study, when autophagy was suppressed in animal sebocytes, a 40% reduction in the proportion of free fatty acids and cholesterol, a five-fold increase in the proportion of FA methyl esters, and a change in sebum composition were demonstrated. Moreover, these changes were consistent with those observed in acne patients [116].

Other studies have examined the relationship between autophagy and acne from a different perspective. It was reported that sebocyte lipogenesis was downregulated when autophagy was induced with the mTOR1 agonist rapamycin, a well-known autophagy inducer. This suggests that autophagy activation could potentially have a positive function in regulating sebocyte lipogenesis and acne development [117]. In addition, according to the results of clinical studies, after the application of the autophagy-activating peptides, decreases in the amount of skin surface lipids (SSL), closed comedones, and trans-epidermal water loss (TEWL) were detected in acne-prone skin [118].

### 6.2. Skin Aging

Cellular senescence is generally induced by DNA damage that involves intracellular (e.g., UVA or UVB irradiation) or extracellular (e.g., reactive oxygen species, ROS) stimulation, and aged cells are distinguished by the senescence-associated secretion of molecules such as proteases, growth factors, and inflammatory cytokines [119,120]. The relationship between aging and autophagy has been studied in various human organs and tissues. Here, we specifically discuss the role of autophagy involved in regulating the function of the skin barrier during aging.

The conditions most often observed in the skin of elderly patients are decreased functional capacity and increased susceptibility of the skin due to exacerbation of skin problems, such as large and small wrinkles, dry skin, itching, dyspigmentation, and tumors. As with other cells, autophagy helps eliminate aged subcellular organelles in aged skin cells. Generally, when autophagy is suppressed, the aging process is intensified through the activation of inflammatory reactions in the skin [121]. In addition, autophagy has been shown to control the functions of dermal fibroblasts, keratinocytes, and melanocytes under UV irradiation or stress conditions known to induce skin aging [122,123].

The effects of autophagy on keratinocytes that form the aged skin barrier have been reported. It was reported that the inhibition of mTORC1 stimulated calcium-induced keratinocyte differentiation [124]. In another study, it was shown that cell death-induced autophagy (CDA) could contribute to the terminal differentiation of skin and skin appendages, including the sebaceous glands [125]. These results suggest that autophagy is closely involved in the process of promoting keratinocyte differentiation in aged skin. Considering that the normal differentiation process of keratinocytes is essential for the formation of the stratum corneum of the skin and the removal of organelles, including the nucleus, is necessary, the regulation of keratinocyte differentiation by autophagy processes is thought to play an important role in maintaining epidermal barrier formation and homeostasis during aging.

Interestingly, one study demonstrated that the UVB-induced autophagy process inhibited epidermal cell death caused by UVB-induced apoptosis through the glycogen synthase kinase signaling pathway [126]. In this study, UVB radiation activated AMPK, a positive regulator of autophagy, through GSK3β inhibition.

## 7. Conclusions and Perspectives

**Figure 1:**
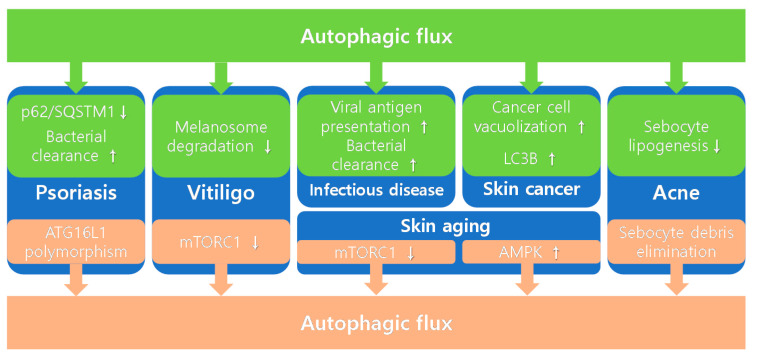
Role of autophagy in skin barrier-related skin diseases.

Autophagy seems to be a fascinating target for novel treatment strategies for various diseases; however, its mechanism is complicated, and the autophagy-related changes vary depending on the disease type. In this review, we focused on the role of autophagy in skin barrier dysfunctions, including autoimmune skin disorders (psoriasis and vitiligo), infectious skin diseases (GAS, candida albicans, and HSV), cancers (SSCC and melanoma), acne, and skin aging. In Figure 1, the role of autophagy in skin barrier-related skin diseases was described. In Table 1, we summarize the autophagy-related changes, consequent physiological changes, and therapeutic effects on each disease. In addition, autophagy-related changes were classified into autophagy-induced changes or autophagy-inducing changes. In most cases, the therapeutic effects of autophagy on skin-related diseases are positive. In psoriasis, p62/SQSTM1 downregulation and increased bacterial clearance lead to positive therapeutic effects. In vitiligo, decreased melanosome degradation and mTORC1 inhibition may influence the positive results. Increased S. pyogenes clearance and enhanced presentation of HSV-1 viral antigen on MHC class I have positive therapeutic effects on infectious diseases. In acne, elimination of sebocytes debris and downregulation of sebocyte lipogenesis can lead to positive outcomes. In skin aging, mTORC1 inhibition and UVB-induced AMPK activation induce positive therapeutic effects. However, when treating tumors, the modulating autophagy strategy should be approached carefully. In the early stages of cancer, autophagy acts as a tumor suppressor; however, in established cancers, autophagy becomes a tumor promotor. In SSCC, autophagy promotes intracellular vacuolization, and this leads to decreased SSCC apoptosis, which has a negative therapeutic effect. In melanoma, autophagy-induced punctate LC3B expression promotes melanoma proliferation and metastasis, leading to a negative therapeutic outcome. The therapeutic materials that mediate autophagy in skin barrier-related skin diseases are summarized in Table 2. Further investigation is needed to clarify the role of autophagy in skin barrier dysfunctions. This will help identify novel potential therapeutic approaches for various skin disorders.

## Figures and Tables

**Table 1:** Involvement of autophagy processes in skin barrier-related skin diseases.

Skin Diseases	Autophagy-Related Changes	Consequent Changes	Therapeutic Effects on Diseases	Refs.
Psoriasis	p62/SQSTM1 downregulation ^a^	Inflammation ↓ Keratinocyte proliferation ↓	Positive	[34]
	Increased bacterial clearance ^a^	Immune response ↓	Positive	[35,36,37]
	ATG16L1 polymorphism	Autophagy flux ↓ Chronic inflammation ↑Cell death ↑	Negative	[38]
Vitiligo	Decreased melanosome degradation ^a^	Melanocyte proliferation ↑	Positive	[62]
	mTORC1 inhibition ^b^	Protection against the metabolic stress on non-lesional melanocytes	Positive	[54]
Infectious disease	Increased S. pyogenes clearance ^a^	Defending against pathogens ↑	Positive	[75,76,77]
	Enhanced presentation of HSV-1 viral antigen on MHC class I ^a^	Degradation of endogenous viral proteins ↑	Positive	[83,84]
Skin cancer	Increased intracellular vacuolization in SSCC ^a^	SSCC apoptosis ↓	Negative	[96]
	Punctate LC3B expression in melanoma ^a^	Melanoma proliferation and metastasis ↑	Negative	[107]
Acne	Sebocytes debris elimination ^b^	Maintaining normal sebaceous gland function	Positive	[115]
	Sebocyte lipogenesis downregulation ^a^	Skin surface lipid, closed comedones, TEWL ↓	Positive	[117,118]
Skin aging	mTORC1 inhibition ^b^	Keratinocyte differentiation ↑	Positive	[124]
	UVB-induced AMPK activation ^b^	Epidermal cell death ↓	Positive	[125]

^a^: Autophagy-induced changes, ^b^: Autophagy-inducing changes. ↓: Inhibition, ↑: Induction.

**Table 2:** Therapeutic materials that mediate autophagy in skin barrier-related skin diseases.

Skin Diseases	Therapeutic Materials	Effects on Autophagy	Consequent Changes	Refs.
Skin squamous cell carcinoma	Chloroquine	↓	Apoptosis ↑	[96,97,98]
Melanoma	Dabrafenib	↓	Antitumor activity ↑	[108]
	Chloroquine	↓	Antitumor activity ↑	[109,110,111,112,113]
Acne	Rapamycin	↑	Lipogenesis ↓, Fatty acid synthesis genes ↓	[117]

↓: Autophagy inhibition, ↑: Autophagy induction.

## Abbreviations

UV	ultraviolet
SC	stratum corneum
ATG	autophagy-related gene
CMA	chaperone-mediated autophagy
Th17	T helper 17
Treg	T regulatory
IL-17A	interleukin-17A
NSV	non-segmental vitiligo
HSP70	heat shock proteins 70
XBP1	X-box binding protein 1
UVRAG	ultraviolet radiation resistance-associated gene
GZMB	granzyme B
GAS	group A Streptococcus
APSGN	acute post-streptococcal glomerulonephritis
ARF	acute rheumatic fever
LC3	microtubule-associated protein light chain 3
NDP52	nuclear dot protein 52
NBR1	neighbor of BRCA1 gene 1
SpeB1	streptococcal pyrogenic exotoxin B
HSV	herpes simplex virus
MHC	major histocompatibility complex (MHC)
ICP34.5	infected cell protein 34.5
EIF2AK2/PKR	eukaryotic translation initiation factor 2-α kinase 2
SSCC	skin squamous cell carcinoma
EGFR	epidermal growth factor receptor
3-MA	3-methyladenine
5-FU	5-fluorouracil
HIF-1α	hypoxia-inducible factor-1α
SSL	skin surface lipids
TEWL	trans-epidermal water loss
ROS	reactive oxygen species
CDA	cell death-induced autophagy
AMPK	AMP-activated protein kinase

## References

[1] Yang Z., Klionsky D.J. (2010). Eaten alive: A history of macroautophagy. Nat. Cell Biol..

[2] Cao W., Li J., Yang K., Cao D. (2021). An overview of autophagy: Mechanism, regulation and research progress. Bull. Cancer.

[3] He L., Zhang J., Zhao J., Ma N., Kim S.W., Qiao S., Ma X. (2018). Autophagy: The last defense against cellular nutritional stress. Adv. Nutr..

[4] Wu H.M., Jiang Z.F., Ding P.S., Shao L.J., Liu R.Y. (2015). Hypoxia-induced autophagy mediates cisplatin resistance in lung cancer cells. Sci. Rep..

[5] Anding A.L., Baehrecke E.H. (2017). Cleaning house: Selective autophagy of organelles. Dev. Cell.

[6] Dainichi T., Hanakawa S., Kabashima K. (2014). Classification of inflammatory skin diseases: A proposal based on the disorders of the three-layered defense systems, barrier, innate immunity and acquired immunity. J. Dermatol. Sci..

[7] Lee S.H., Jeong S.K., Ahn S.K. (2006). An update of the defensive barrier function of skin. Yonsei Med. J..

[8] Salmon J.K., Armstrong C.A., Ansel J.C. (1994). The Skin as an Immune Organ. West. J. Med..

[9] Zimmerman A., Bai L., Ginty D.D. (2014). The gentle touch receptors of mammalian skin. Science.

[10] Woo W.M. (2019). Skin structure and biology. Imaging Technol. Transdermal Deliv. Ski. Disord..

[11] Boulais N., Pereira U., Lebonvallet N., Misery L. (2007). The whole epidermis as the forefront of the sensory system. Exp. Dermatol..

[12] Khavkin J., Ellis D.A.F. (2011). Aging Skin: Histology, Physiology, and Pathology. Facial Plast. Surg. Clin..

[13] Yu T., Zuber J., Li J. (2015). Targeting autophagy in skin diseases. J. Mol. Med..

[14] Mizushima N., Komatsu M. (2011). Autophagy: Renovation of cells and tissues. Cell.

[15] Levine B., Mizushima N., Virgin H.W. (2011). Autophagy in immunity and inflammation. Nature.

[16] Levine B., Kroemer G. (2019). Biological Functions of Autophagy Genes: A Disease Perspective. Cell.

[17] Fujiwara Y., Wada K., Kabuta T. (2017). Lysosomal degradation of intracellular nucleic acids-multiple autophagic pathways. J. Biochem..

[18] Schuck S. (2020). Microautophagy—Distinct molecular mechanisms handle cargoes of many sizes. J. Cell Sci..

[19] Kaushik S., Cuervo A.M. (2018). The coming of age of chaperone-mediated autophagy. Nat. Rev. Mol. Cell Biol..

[20] Gatica D., Lahiri V., Klionsky D.J. (2018). Cargo recognition and degradation by selective autophagy. Nat. Cell Biol..

[21] Pohl C., Dikic I. (2019). Cellular quality control by the ubiquitin-proteasome system and autophagy. Science.

[22] Escobar K.A., Cole N.H., Mermier C.M., VanDusseldorp T.A. (2019). Autophagy and aging: Maintaining the proteome through exercise and caloric restriction. Aging Cell.

[23] Mizushima N., Levine B. (2010). Autophagy in mammalian development and differentiation. Nat. Cell Biol..

[24] Clarke A.J., Simon A.K. (2019). Autophagy in the renewal, differentiation and homeostasis of immune cells. Nat. Rev. Immunol..

[25] Deretic V., Levine B. (2018). Autophagy balances inflammation in innate immunity. Autophagy.

[26] Matsuzawa-Ishimoto Y., Hwang S., Cadwell K. (2018). Autophagy and Inflammation. Annu. Rev. Immunol..

[27] Poillet-Perez L., White E. (2019). Role of tumor and host autophagy in cancer metabolism. Genes Dev..

[28] Wu D.J., Adamopoulos I.E. (2017). Autophagy and autoimmunity. Clin. Immunol..

[29] Griffiths C.E., Barker J.N. (2007). Pathogenesis and clinical features of psoriasis. Lancet.

[30] Rendon A., Schäkel K. (2019). Psoriasis Pathogenesis and Treatment. Int. J. Mol. Sci..

[31] Chang J., Choi M.S. (2020). A Molecular Perspective on the Potential Benefits of Metformin for the Treatment of Inflammatory Skin Disorders. Int. J. Mol. Sci..

[32] Svendsen M.T., Jeyabalan J., Andersen K.E., Andersen F., Johannessen H., Tiedemann M., Jeyabalan J., Andersen K.E. (2016). Worldwide utilization of topical remedies in treatment of psoriasis: A systematic review. J. Dermatolog. Treat..

[33] Iskandar I.Y.K.D., Parisi R.D., Griffiths C.E.M., Ashcroft D.M., Psoriasis G. (2021). Systematic review examining changes over time and variation in the incidence and prevalence of psoriasis by age and gender. Br. J. Dermatol..

[34] Lee H., Shin D., Yuk J., Shi G., Choi D., Lee S., Huang S.M., Kim J., Kim C.D., Lee J. (2011). Autophagy Negatively Regulates Keratinocyte Inflammatory Responses via Scaffolding Protein p62/SQSTM1. J. Immunol..

[35] Fry L., Baker B.S., Powles A.V., Fahlen A., Engstrand L. (2013). Is chronic plaque psoriasis triggered by microbiota in the skin?. Br. J. Dermatol..

[36] De Jesús-Gil C., Sans-de San Nicolás L., Ruiz-Romeu E., Ferran M., Soria-Martinez L., Chiriac A., Celada A., Pujol R.M., Santamaria-Babí L.F. (2020). Specific IgA and CLA+ T-Cell IL-17 Response to Streptococcus pyogenes in Psoriasis. J. Investig. Dermatol..

[37] Khaleel R.A., Munaf H., Zalzala W., Rashid Y. (2021). Role of Autophagy in Psoriasis. Eur. J. Mol. Clin. Med..

[38] Douroudis K., Kingo K., Traits T., Reimann E., Raud K., Rätsep R., Mössner S.R. (2011). Polymorphisms in the ATG16L1 Gene are Associated with Psoriasis Vulgaris No significant deviation from the Hardy-Weinberg. Acta Derm. Venereol..

[39] Mizushima N., Kuma A., Kobayashi Y., Yamamoto A., Matsubae M. (2003). Mouse Apg16L, a novel WD-repeat protein, targets to the autophagic isolation membrane with the Apg12- Apg5 conjugate. J. Cell Sci..

[40] Guo Y., Zhang X., Wu T., Hu X., Su J., Chen X. (2019). Autophagy in Skin Diseases. Dermatology.

[41] Zaba L.C., Cardinale I., Gilleaudeau P., Sullivan-Whalen M., Fariñas M.S., Fuentes-Duculan J., Novitskaya I., Khatcherian A., Bluth M.J., Lowes M.A. (2007). Amelioration of epidermal hyperplasia by TNF inhibition is associated with reduced Th17 responses. J. Exp. Med..

[42] Sugiyama H., Gyulai R., Toichi E., Garaczi E., Shimada S., Stevens S.R., McCormick T.S., Cooper K.D. (2005). Dysfunctional Blood and Target Tissue CD4 + CD25 high Regulatory T Cells in Psoriasis: Mechanism Underlying Unrestrained Pathogenic Effector T Cell Proliferation. J. Immunol..

[43] Zaba L.C., Fuentes-Duculan J., Eungdamrong N.J., Abello M.V., Novitskaya I., Pierson K.C., Gonzalez J., Krueger J.G., Lowes M.A. (2009). Psoriasis is characterized by accumulation of immunostimulatory and Th1/Th17 cell-polarizing myeloid dendritic cells. J. Investig. Dermatol..

[44] Furue M., Furue K., Tsuji G., Nakahara T. (2020). Interleukin-17A and keratinocytes in psoriasis. Int. J. Mol. Sci..

[45] Li B., Huang L., Lv P., Li X., Liu G., Chen Y., Wang Z., Qian X., Shen Y., Li Y. (2020). The role of Th17 cells in psoriasis. Immunol. Res..

[46] Afzali B., Lombardi G., Lechler R.I., Lord G.M. (2007). The role of T helper 17 (Th17) and regulatory T cells (Treg) in human organ transplantation and autoimmune disease. Clin. Exp. Immunol..

[47] Arican O., Aral M., Sasmaz S., Ciragil P. (2005). Serum levels of TNF-α, IFN-γ, IL-6, IL-8, IL-12, IL-17, and IL-18 in patients with active psoriasis and correlation with disease severity. Mediators Inflamm..

[48] Ureshino H., Kamachi K., Kimura S. (2019). Mogamulizumab for the Treatment of Adult T-cell Leukemia/Lymphoma. Clin. Lymphoma Myeloma Leuk..

[49] Nussbaum L., Chen Y.L., Ogg G.S. (2021). Role of regulatory T cells in psoriasis pathogenesis and treatment. Br. J. Dermatol..

[50] Zhang L., Li Y., Yang X., Wei J., Zhou S., Zhao Z., Cheng J., Duan H., Jia T., Lei Q. (2016). Characterization of Th17 and FoxP3+ Treg Cells in Paediatric Psoriasis Patients. Scand. J. Immunol..

[51] Soler D.C., Sugiyama H., Young A.B., Massari J.V., McCormick T.S., Cooper K.D. (2013). Psoriasis patients exhibit impairment of the high potency CCR5+ T regulatory cell subset. Clin. Immunol..

[52] Lee S.K., Park M.J., Jhun J.Y., Beak J.A., Choi J.W., Rye J.Y., Jang J.W., Bae S.H., Yoon S.K., Choi H.J. (2021). Combination Treatment With Metformin and Tacrolimus Improves Systemic Immune Cellular Homeostasis by Modulating Treg and Th17 Imbalance. Front. Immunol..

[53] Samaka R.M., Basha M.A., Zahran A.M. (2018). Role of beclin 1 and autophagy in vitiligo. Menoufia Med. J..

[54] Bastonini E., Kovacs D., Raffa S., delle Macchie M., Pacifico A., Iacovelli P., Torrisi M.R., Picardo M. (2021). A protective role for autophagy in vitiligo. Cell Death Dis..

[55] Ghasemloo S., Gauthier Y., Ghalamkarpour F. (2019). Evaluation of using fractional CO_2_ laser plus NB-UVB versus NB-UVB alone in inducing marginal repigmentation of vitiligo lesions. J. Dermatolog. Treat..

[56] Bzioueche H., Sjödin K.S., West C.E., Khemis A., Rocchi S., Passeron T., Tulic M.K. (2021). Analysis of Matched Skin and Gut Microbiome of Vitiligo Patients Reveals Deep skin Dysbiosis: Link with Mitochondrial and Immune Changes. J. Investig. Dermatol..

[57] Iannella G., Greco A., Didona D., Didona B., Granata G., Manno A., Pasquariello B., Magliulo G. (2016). Vitiligo: Pathogenesis, clinical variants and treatment approaches. Autoimmun. Rev..

[58] Taïeb A., Picardo M. (2007). The definition and assessment of vitiligo: A consensus report of the Vitiligo European Task Force. Pigment Cell Res..

[59] Jeong K.H., Kim S.K., Seo J.K., Shin M.K., Lee M.H. (2021). Association of GZMB polymorphisms and susceptibility to non-segmental vitiligo in a Korean population. Sci. Rep..

[60] Harris J.E. (2016). Cellular stress and innate inflammation in organ-specific autoimmunity: Lessons learned from vitiligo. Immunol. Rev..

[61] Sil P., Wong S.W., Martinez J. (2018). More than skin deep: Autophagy is vital for skin barrier function. Front. Immunol..

[62] Murase D., Hachiya A., Takano K., Hicks R., Visscher M.O., Kitahara T., Hase T., Takema Y., Yoshimori T. (2013). Autophagy has a significant role in determining skin color by regulating melanosome degradation in keratinocytes. J. Investig. Dermatol..

[63] Kalie E., Razi M., Tooze S.A. (2013). ULK1 Regulates Melanin Levels in MNT-1 Cells Independently of mTORC1. PLoS ONE.

[64] Jeong T.J., Shin M.K., Uhm Y.K., Kim H.J., Chung J.H., Lee M.H. (2010). Association of UVRAG polymorphisms with susceptibility to non-segmental vitiligo in a Korean sample. Exp. Dermatol..

[65] Barnett T.C., Bowen A.C., Carapetis J.R. (2019). The fall and rise of Group A Streptococcus diseases. Epidemiol. Infect..

[66] Cannon J.W., Jack S., Wu Y., Zhang J., Baker M.G., Geelhoed E., Fraser J., Carapetis J.R. (2018). An economic case for a vaccine to prevent group A streptococcus skin infections. Vaccine.

[67] Zühlke L.J., Beaton A., Engel M.E., Hugo-Hamman C.T., Karthikeyan G., Katzenellenbogen J.M., Ntusi N., Ralph A.P., Saxena A., Smeesters P.R. (2017). Group A streptococcus, acute rheumatic fever and rheumatic heart disease: Epidemiology and clinical considerations. Curr. Treat. Options Cardiovasc. Med..

[68] Walker M.J., Barnett T.C., McArthur J.D., Cole J.N., Gillen C.M., Henningham A., Sriprakash K.S., Sanderson-Smith M.L., Nizet V. (2014). Disease manifestations and pathogenic mechanisms of group A Streptococcus. Clin. Microbiol. Rev..

[69] Nakagawa I., Amano A., Mizushima N., Yamamoto A., Yamaguchi H., Kamimoto T., Nara A., Funao J., Nakata M., Tsuda K. (2004). Autophagy defends cells against invading group A Streptococcus. Science.

[70] Zheng Y.T., Shahnazari S., Brech A., Lamark T., Johansen T., Brumell J.H. (2009). The adaptor protein p62/SQSTM1 targets invading bacteria to the autophagy pathway. J. Immunol..

[71] Fan S., Wu K., Zhao M., Zhu E., Ma S., Chen Y., Ding H., Yi L., Zhao M., Chen J. (2020). The Role of Autophagy and Autophagy Receptor NDP52 in Microbial Infections. Int. J. Mol. Sci..

[72] Von Muhlinen N., Thurston T., Ryzhakov G., Bloor S., Randow F. (2010). NDP52, a novel autophagy receptor for ubiquitin-decorated cytosolic bacteria. Autophagy.

[73] Itakura E., Mizushima N. (2011). p62 Targeting to the autophagosome formation site requires self-oligomerization but not LC3 binding. J. Cell Biol..

[74] Lu S.-L., Kuo C.-F., Chen H.-W., Yang Y.-S., Liu C.-C., Anderson R., Wu J.-J., Lin Y.-S. (2015). Insufficient acidification of autophagosomes facilitates group A streptococcus survival and growth in endothelial cells. MBio.

[75] Wang J., Meng M., Li M., Guan X., Liu J., Gao X., Sun Q., Li J., Ma C., Wei L. (2020). Integrin α5β1, as a Receptor of Fibronectin, Binds the FbaA Protein of Group A Streptococcus To Initiate Autophagy during Infection. MBio.

[76] Hsieh C.L., Hsieh S.Y., Huang H.M., Lu S.L., Omori H., Zheng P.X., Ho Y.N., Cheng Y.L., Lin Y.S., Chiang-Ni C. (2020). Nicotinamide Increases Intracellular NAD+ Content to Enhance Autophagy-Mediated Group A Streptococcal Clearance in Endothelial Cells. Front. Microbiol..

[77] Nakajima K., Nozawa T., Minowa-Nozawa A., Toh H., Yamada S., Aikawa C., Nakagawa I. (2019). RAB30 regulates PI4KB (phosphatidylinositol 4-kinase beta)-dependent autophagy against group A Streptococcus. Autophagy.

[78] Barnett T.C., Liebl D., Seymour L.M., Gillen C.M., Lim J.Y., Larock C.N., Davies M.R., Schulz B.L., Nizet V., Teasdale R.D. (2013). The globally disseminated M1T1 clone of group a streptococcus evades autophagy for intracellular replication. Cell Host Microbe.

[79] Fatahzadeh M., Schwartz R.A. (2007). Human herpes simplex virus infections: Epidemiology, pathogenesis, symptomatology, diagnosis, and management. J. Am. Acad. Dermatol..

[80] Wang J., Yuan S., Zhu D., Tang H., Wang N., Chen W., Gao Q., Li Y., Wang J., Liu H. (2018). Structure of the herpes simplex virus type 2 C-capsid with capsid-vertex-specific component. Nat. Commun..

[81] Lafaille F.G., Pessach I.M., Zhang S.-Y., Ciancanelli M.J., Herman M., Abhyankar A., Ying S.-W., Keros S., Goldstein P.A., Mostoslavsky G. (2012). Impaired intrinsic immunity to HSV-1 in human iPSC-derived TLR3-deficient CNS cells. Nature.

[82] Lussignol M., Esclatine A. (2017). Herpesvirus and Autophagy: “All Right, Everybody Be Cool, This Is a Robbery!”. Viruses.

[83] O’Connell D., Liang C. (2016). Autophagy interaction with herpes simplex virus type-1 infection. Autophagy.

[84] English L., Chemali M., Duron J., Rondeau C., Laplante A., Gingras D., Alexander D., Leib D., Norbury C., Lippé R. (2009). Autophagy enhances the presentation of endogenous viral antigens on MHC class I molecules during HSV-1 infection. Nat. Immunol..

[85] Lee H.K., Mattei L.M., Steinberg B.E., Alberts P., Lee Y.H., Chervonsky A., Mizushima N., Grinstein S., Iwasaki A. (2010). In vivo requirement for Atg5 in antigen presentation by dendritic cells. Immunity.

[86] Leib D.A., Alexander D.E., Cox D., Yin J., Ferguson T.A. (2009). Interaction of ICP34. 5 with Beclin 1 modulates herpes simplex virus type 1 pathogenesis through control of CD4+ T-cell responses. J. Virol..

[87] Lussignol M., Queval C., Bernet-Camard M.-F., Cotte-Laffitte J., Beau I., Codogno P., Esclatine A. (2013). The herpes simplex virus 1 Us11 protein inhibits autophagy through its interaction with the protein kinase PKR. J. Virol..

[88] Nicola A.M., Albuquerque P., Martinez L.R., Dal-Rosso R.A., Saylor C., De Jesus M., Nosanchuk J.D., Casadevall A. (2012). Macrophage autophagy in immunity to Cryptococcus neoformans and Candida albicans. Infect. Immun..

[89] Ma T., Yu Q., Ma C., Mao X., Liu Y., Peng X., Li M. (2020). Role of the inositol polyphosphate kinase Vip1 in autophagy and pathogenesis in Candida albicans. Future Microbiol..

[90] White E. (2012). Deconvoluting the context-dependent role for autophagy in cancer. Nat. Rev. Cancer.

[91] Singh S.S., Vats S., Chia A.Y.Q., Tan T.Z., Deng S., Ong M.S., Arfuso F., Yap C.T., Goh B.C., Sethi G. (2018). Dual role of autophagy in hallmarks of cancer. Oncogene.

[92] Yun C.W., Lee S.H. (2018). The roles of autophagy in cancer. Int. J. Mol. Sci..

[93] Leiter U., Keim U., Garbe C., Reichrath J. (2020). Epidemiology of Skin Cancer: Update 2019. Sunlight, Vitamin D and Skin Cancer.

[94] Lai V., Cranwell W., Sinclair R. (2018). Epidemiology of skin cancer in the mature patient. Clin. Dermatol..

[95] Gong Z., Ji J., Yao J., Ji J., Jiang Y., Gao G., Feng Z. (2020). The anti-skin squamous cell carcinoma cell activity by the SphK1- targeting microRNA-6784. Aging Albany.

[96] Verschooten L., Barrette K., van Kelst S., Rubio Romero N., Proby C., de Vos R., Agostinis P., Garmyn M. (2012). Autophagy Inhibitor Chloroquine Enhanced the Cell Death Inducing Effect of the Flavonoid Luteolin in Metastatic Squamous Cell Carcinoma Cells. PLoS ONE.

[97] Ou C., Liu H., Ding Z., Zhou L. (2019). Chloroquine promotes gefitinib-induced apoptosis by inhibiting protective autophagy in cutaneous squamous cell carcinoma. Mol. Med. Rep..

[98] Wang J., Wang C., Hu X., Yu C., Zhou L., Ding Z., Zhou M. (2019). Gefitinib-mediated apoptosis is enhanced via inhibition of autophagy by chloroquine diphosphate in cutaneous squamous cell carcinoma cells. Oncol. Lett..

[99] Zhang L., Zhang J., Chen L., Wang J. (2015). Autophagy in human skin squamous cell carcinoma: Inhibition by 3-MA enhances the effect of 5-FU-induced chemotherapy sensitivity. Oncol. Rep..

[100] Ndoye A., Weeraratna A.T. (2016). Autophagy—An emerging target for melanoma therapy. F1000Research.

[101] Berk-Krauss J., Stein J.A., Weber J., Polsky D., Geller A.C. (2020). New systematic therapies and trends in cutaneous melanoma deaths among US whites, 1986–2016. Am. J. Public Health.

[102] Tomic T., Botton T., Cerezo M., Robert G., Luciano F., Puissant A., Gounon P., Allegra M., Bertolotto C., Bereder J.M. (2011). Metformin inhibits melanoma development through autophagy and apoptosis mechanisms. Cell Death Dis..

[103] Yajima I., Kumasaka M.Y., Thang N.D., Goto Y., Takeda K., Yamanoshita O., Iida M., Ohgami N., Tamura H., Kawamoto Y. (2012). RAS/RAF/MEK/ERK and PI3K/PTEN/AKT signaling in malignant melanoma progression and therapy. Dermatol. Res. Pract..

[104] Gray-Schopfer V.C., Da Rocha Dias S., Marais R. (2005). The role of B-RAF in melanoma. Cancer Metastasis Rev..

[105] Hodi F.S., O’Day S.J., McDermott D.F., Weber R.W., Sosman J.A., Haanen J.B., Gonzalez R., Robert C., Schadendorf D., Hassel J.C. (2010). Improved Survival with Ipilimumab in Patients with Metastatic Melanoma. N. Engl. J. Med..

[106] Flaherty K.T., Puzanov I., Kim K.B., Ribas A., McArthur G.A., Sosman J.A., O’Dwyer P.J., Lee R.J., Grippo J.F., Nolop K. (2010). Inhibition of Mutated, Activated BRAF in Metastatic Melanoma. N. Engl. J. Med..

[107] Lazova R., Camp R.L., Klump V., Siddiqui S.F., Amaravadi R.K., Pawelek J.M. (2012). Punctate LC3B expression is a common feature of solid tumors and associated with proliferation, metastasis, and poor outcome. Clin. Cancer Res..

[108] Xie X., Koh J.Y., Price S., White E., Mehnert J.M. (2015). Atg7 overcomes senescence and promotes growth of BRAFV600E_driven melanoma. Cancer Discov..

[109] INOUE S., HASEGAWA K., ITO S., WAKAMATSU K., FUJITA K. (1993). Antimelanoma Activity of Chloroquine, an Antimalarial Agent with High Affinity for Melanin. Pigment Cell Res..

[110] Harhaji-Trajkovic L., Arsikin K., Kravic-Stevovic T., Petricevic S., Tovilovic G., Pantovic A., Zogovic N., Ristic B., Janjetovic K., Bumbasirevic V. (2012). Chloroquine-mediated lysosomal dysfunction enhances the anticancer effect of nutrient deprivation. Pharm. Res..

[111] Egger M.E., Huang J.S., Yin W., McMasters K.M., McNally L.R. (2013). Inhibition of autophagy with chloroquine is effective in melanoma. J. Surg. Res..

[112] Rangwala R., Leone R., Chang Y.C., Fecher L.A., Schuchter L.M., Kramer A., Tan K.S., Heitjan D.F., Rodgers G., Gallagher M. (2014). Phase I trial of hydroxychloroquine with dose-intense temozolomide in patients with advanced solid tumors and melanoma. Autophagy.

[113] Rangwala R., Chang Y.C., Hu J., Algazy K.M., Evans T.L., Fecher L.A., Schuchter L.M., Torigian D.A., Panosian J.T., Troxel A.B. (2014). Combined MTOR and autophagy inhibition: Phase I trial of hydroxychloroquine and temsirolimus in patients with advanced solid tumors and melanoma. Autophagy.

[114] Williams H.C., Dellavalle R.P., Garner S. (2012). Acne vulgaris. Lancet.

[115] Rossiter H., Stübiger G., Gröger M., König U., Gruber F., Sukseree S., Mlitz V., Buchberger M., Oskolkova O., Bochkov V. (2018). Inactivation of autophagy leads to changes in sebaceous gland morphology and function. Exp. Dermatol..

[116] Zouboulis C.C., Jourdan E., Picardo M. (2014). Acne is an inflammatory disease and alterations of sebum composition initiate acne lesions. J. Eur. Acad. Dermatology Venereol..

[117] Seo S.H., Jung J.Y., Park K., Hossini A.M., Zouboulis C.C., Lee S.E. (2020). Autophagy regulates lipid production and contributes to the sebosuppressive effect of retinoic acid in human SZ95 sebocytes. J. Dermatol. Sci..

[118] Lee Y., Shin K., Shin K.O., Yoon S., Jung J., Hwang E., Chung H.J., Hossini A.M., Zouboulis C.C., Baek M.J. (2021). Topical application of autophagy-activating peptide improved skin barrier function and reduced acne symptoms in acne-prone skin. J. Cosmet. Dermatol..

[119] Galbiati A., Beauséjour C., d’Adda di Fagagna F. (2017). A novel single-cell method provides direct evidence of persistent DNA damage in senescent cells and aged mammalian tissues. Aging Cell.

[120] Malaquin N., Martinez A., Rodier F. (2016). Keeping the senescence secretome under control: Molecular reins on the senescence-associated secretory phenotype. Exp. Gerontol..

[121] Chen R.J., Lee Y.H., Yeh Y.L., Wang Y.J., Wang B.J. (2016). The roles of autophagy and the inflammasome during environmental stress-triggered skin inflammation. Int. J. Mol. Sci..

[122] Jeong D., Qomaladewi N.P., Lee J., Park S.H., Cho J.Y. (2020). The Role of Autophagy in Skin Fibroblasts, Keratinocytes, Melanocytes, and Epidermal Stem Cells. J. Investig. Dermatol..

[123] Gu Y., Han J., Jiang C., Zhang Y. (2020). Biomarkers, oxidative stress and autophagy in skin aging. Ageing Res. Rev..

[124] Mahanty S., Dakappa S.S., Shariff R., Patel S., Swamy M.M., Majumdar A., Gangi Setty S.R. (2019). Keratinocyte differentiation promotes ER stress-dependent lysosome biogenesis. Cell Death Dis..

[125] Koenig U., Robenek H., Barresi C., Brandstetter M., Resch G.P., Gröger M., Pap T., Hartmann C. (2020). Cell death induced autophagy contributes to terminal differentiation of skin and skin appendages. Autophagy.

[126] Yang Y., Wang H., Wang S., Xu M., Liu M., Liao M., Frank J.A., Adhikari S., Bower K.A., Shi X. (2012). GSK3β signaling is involved in ultraviolet B-induced activation of autophagy in epidermal cells. Int. J. Oncol..

